# Epidemiological study on ticks and tick-borne pathogens in cape hare (*Lepus capensis*) in Algeria

**DOI:** 10.3389/fvets.2026.1936427

**Published:** 2026-09-11

**Authors:** Abderrazek Bendrimia, Faouzi Matallah, Francesco Defilippo, Sara Rigamonti, Paola Prati, Maria Sampieri, Patrizia Cavadini, Antonio Lavazza, Tiziana Trogu, Mohamed Besbaci, Sofiane Bensefia, Hamza Khaled, Madjid Akkou

**Affiliations:** 1 Laboratory EHPREC, Department of Veterinary Sciences, Faculty of Natural and Life Sciences, University Chadli Ben Djedid, El Tarf, Algeria; 2 Istituto Zooprofilattico Sperimentale della Lombardia e dell’Emilia Romagna, Brescia, Italy; 3 LAB, Veterinary Institute, Saad Dahleb University, Blida, Algeria; 4 Department of Agricultural Sciences, Faculty of Natural and Life Sciences, University Mohamed El Bachir El Ibrahimi, Bordj Bou Arreridj, Algeria

**Keywords:** Algeria, *Lepus capensis*, PCR, ticks, zoonotic pathogens

## Abstract

**Introduction:**

The Cape hare (*L. capensis*) is one of the most common game species in Algeria, although its populations have declined in recent years. This wild lagomorph may host ticks, which act as vectors for a range of zoonotic pathogens. The purpose of this study is to fill the gaps in current knowledge regarding the presence of ticks in Algerian cape hare species and better understand the potential implications for public health.

**Methods:**

During the 2023–2024 hunting seasons in the High Plateaus of Algeria, a total of 47 lagomorphs (44 Cape hares and 3 wild rabbits) were collected from 20 sites and examined for tick infestation. Ticks were identified using both morphological and molecular approaches. The presence of bacterial pathogens (*Borrelia* spp., *Rickettsia* spp., *Anaplasma* spp., *Coxiella* spp., *Ehrlichia* spp.) and viral agents (Tick Borne Encephalites Virus, Crimean Congo Haemorragic Fever, West Nile Virus, Usutu virus, and phleboviruses) was assessed using quantitative real-time PCR on 56 ticks pools.

**Results:**

Twenty-two hares (50%) but no rabbits were infested with ticks. A total of 165 ticks were collected, predominantly *Rhipicephalus sanguineus* sensu lato (94.5%), followed by *Ixodes hexagonus* (5.5%). Adult ticks were the most abundant life stage, followed by nymphs, while larvae were rarely observed. Seasonal analysis showed a peak infestation in September (100% prevalence, mean intensity 11.57 ± 22.37 ticks/animal), followed by a decline in October (40.9%, 2.27 ± 3.77 ticks/animal) and November (46.2%, 1.82 ± 3.52 ticks/animal). No ticks were detected in December. Molecular screening revealed seven zoonotic *Rickettsia* species in *R. sanguineus*, with *R. aeschlimannii* being most prevalent (19.6%). Additionally, *Borrelia afzelii* (3.6%) and *B. burgdorferi* sensu stricto (1.8%) were detected, with a total prevalence of 0.7% (4 positive pools). All ticks were negative for *Anaplasma* spp., *Coxiella* spp., *Ehrlichia* spp., and screened viruses.

**Conclusion:**

*R. sanguineus* is the predominant tick species infesting Cape hares in Algeria. The detection of multiple zoonotic pathogens highlights the potential role of this host in the epidemiology of tick-borne diseases and underscores the need for expanded surveillance in wild lagomorph populations within a One Health framework.

## 1 Introduction

The Cape hare (*L. capensis*) is one of the most widespread wild lagomorphs in Algeria, serving both as an ecologically important prey species and a target for hunting activities (1). Despite the role it plays in the country’s economy, little attention has ever been paid to the health monitoring of this species, giving priority to issues more closely linked to the country’s economic and social aspects. Knowledge was often limited to a few parasitological studies—and only recently concerned tularemia (2)—without, however, ever establishing targeted active surveillance plans. This highlights the lack of systematic surveillance data and the need to better define the role of this species in the maintenance and transmission of pathogens within a ‘One Health’ approach. Indeed, this species may act as a reservoir for zoonotic pathogens transmitted by ticks (3–5), a topic that is attracting increasing interest from the scientific community.

Globally, tick-borne diseases have risen considerably over recent decades (6, 7). However, data on tick infestation in Cape hares in Algeria are limited. To date, only a few studies have documented tick species associated with wild lagomorphs in north-central Algeria, including *Riphicephalus Sanguineus* sensu lato, *Rhipicephalus pusillus*, *Ixodes ricinus*, and *Hyalomma* spp. (2). These ticks are recognised vectors of several zoonotic pathogens, including agents responsible for tularemia (1, 2).

*Rhipicephalus sanguineus*, commonly associated with dogs, is a highly adaptable species capable of parasitising a wide range of hosts, including hares, wild carnivores, and hedgehogs (8–10). Although primarily endophilic, it can also survive in outdoor environments. This tick is a known vector of several pathogens, including *Babesia canis*, *Ehrlichia canis*, and multiple *Rickettsia* species, such as *R. conorii* and *R. rickettsii* (11–14). In North Africa, several Rickettsia species of public health concern have been detected in both ticks and humans, including *R. aeschlimannii*, *R. massiliae* and *R. sibirica* (15–19). In Algeria, an increasing number of cases of Mediterranean spotted fever have been reported, with tick bites documented in 50.3% of patients (20). Moreover, *R. aeschlimannii* has been identified in two human cases (20).

The *Borrelia burgdorferi* sensu lato group, the causative agent of Lyme borreliosis (LB), is the most common tick-borne infectious disease (21). *Ixodes ricinus*, the primary vector in Europe, feeds on a broad range of hosts, including reptiles, birds, and mammals, from small insectivores to large ungulates (22). A study in Norway detected *B. burgdorferi* sl in internal organs and identified *B. afzelii*, *B. burgdorferi* sensu stricto, and *Borrelia* sp. SV1 in skin samples, as well as in *I. ricinus* ticks collected from mountain hares (*Lepus timidus*), suggesting a potential role of hares in Lyme borreliosis ecology (23). Additional studies in Europe have reported the presence of *Borrelia* spp. in *I. ricinus* and *I. hexagonus* ticks collected from hedgehogs (24). In Algeria, recent PCR-based studies have reported relapsing fever borreliae, including *Borrelia hispanica* and *Borrelia cf. turicatae* in soft ticks (25), as well as *Borrelia* spp. in *I. ricinus* ticks collected from cattle (26). Despite the wide distribution of Cape hares, information on their tick infestations and associated pathogens remains limited. Therefore, understanding their role in the ecology of tick-borne diseases is essential.

This study aimed to characterise the tick species infesting Cape hares in Algeria using morphological and molecular approaches and to assess seasonal infestation patterns. Moreover, since ticks are recognised as reservoirs of numerous bacterial and viral pathogens, investigating the co-occurrence of these pathogens helps to better understand their ecology, identify any coinfections, and assess more accurately the risks they pose to animal and human health. From a broader epidemiological perspective, wildlife may play an important role in maintaining tick populations and facilitating the circulation of tick-borne pathogens between natural, domestic, and human environments. This highlights the importance of adopting a One Health approach, recognising the interconnectedness of human, animal, and environmental health. In this context, investigating tick–host–pathogen associations in Cape hares can provide valuable information on pathogen circulation at the wildlife–animal–human interface and contribute to improved surveillance and risk assessment of tick-borne diseases.

## 2 Materials and methods

### 2.1 Study area

**Figure 1:**
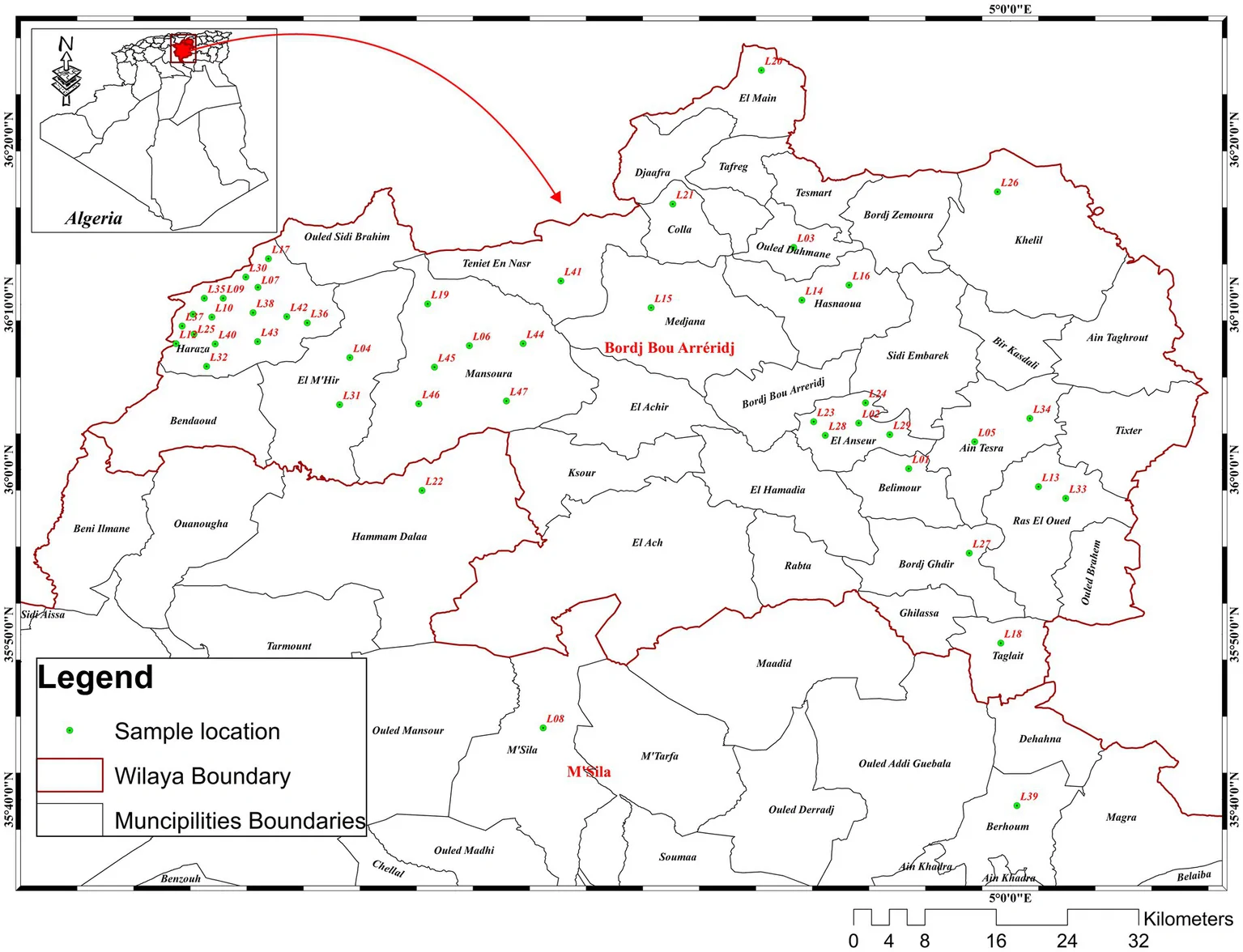
Map of the Algerian provinces Bordj Bou Arreridj and M’sila, showing the sampling location of hunted wild lagomorphs.

The study was conducted in two regions of northern Algeria: Bordj-Bou-Arreridj (36°04′16.29″N, 4°45′31.69″E), comprising 17 sampling sites, and M’sila (35°42′07.00″N, 4°32′50.00″E) with three sampling sites (Figure 1). Bordj-Bou-Arreridj is characterised by a semi-arid bioclimate with hot summers. July is the warmest month, with an average maximum temperature of 39.5 °C, while winters are relatively cold, with January showing the lowest average minimum temperature (−3.45 °C). The altitude ranges from 700 to 1741 m.s.l., and the average annual rainfall is 519 mm (27). M’sila is a steppe region surrounded by mountains, with a semi-arid, continental climate. The mean annual temperature is 19.5 °C. Altitudes range from 400 to 1,000 m above sea level, and the average annual rainfall is 298.18 mm (28).

### 2.2 Animal sampling

In Algeria, a regulatory framework sets out specific agreements between hunters and hunting associations governing the collection of biological samples from hunted animals, which are required for the surveillance programme. Between the 2023 and 2024 hunting seasons, a total of 44 Cape hares and 3 wild rabbits were collected from 20 sites. The sample size was therefore primarily determined by the availability of free-ranging wildlife during the study period. Each animal/carcass was packed in an individual plastic bag, frozen, and transferred to the laboratory, where it was examined according to a standardised protocol (29). The fur, skin, and head were inspected macroscopically for the presence of ectoparasites. For each animal, the following data were recorded (Table 1; Supplementary material): identification number, sampling date, location, sex, body weight, and body length. Age was determined using Stroh’s method: individuals were classified as juveniles when the Stroh’s tubercle was palpable on the distal ulna and as adults when the bone was smooth and fused (30). Hares were identified as Cape hares based on phenotypic and morphometric characteristics.

**Table 1:** Data of the examined lagomorphs during the period 2023/2024.

Region	Examined hares	Sex		Age	
		Male	Female	Adult	Juvenile
Bordj Bou Arreridj	44	27	17	23	21
M’sila	3	3	0	2	1
Total	47	30	17	25	22

### 2.3 Tick sampling and identification

Ticks were carefully removed using fine forceps and stored individually in sterile plastic tubes containing 70% ethanol until further analysis. Ticks were sorted into pools (from 1 to 6 specimens) by hare, site, and sampling date. They were morphologically identified at the species level with a stereomicroscope using taxonomic identification keys (31–37).

Among the tick species identified, *R. sanguineus* sensu lato (sl) needs particular attention. The *R. sanguineus* group comprises multiple morphologically similar yet phylogenetically distinct species (38), and their accurate identification by morphological keys alone is often unreliable, particularly for immature stages. Taxonomy within this group remains controversial, compounded by the limited availability of reference DNA sequences in public databases (39). For these reasons, specimens belonging to the *R. sanguineus* complex were selected for identification by matrix-assisted laser desorption/ionisation time-of-flight mass spectrometry (MALDI-TOF MS), with the aim of generating reference mass spectra for eventual *Rh. pusillus* species; a tick species not represented species in existing proteomic databases.

Specimens with ambiguous morphological features were further analysed using MALDI-TOF MS, following a previously described protocol (40, 41). Briefly, four legs from each tick were excised using a sterile surgical blade and transferred into a 1.5 mL Eppendorf tube. The legs, previously stored at −20 °C, were directly homogenised and processed for MALDI-TOF MS analysis.

The remaining half of the four legs was preserved at −20 °C as a backup sample for potential DNA extraction and molecular identification if needed.

### 2.4 Molecular analysis of pathogens

The ticks were homogenised in phosphate-buffered saline (PBS) with Tissue Lyser II (Qiagen). DNA was extracted from 200 μL of homogenate through the IndiMag Pathogen kit (Indical Biosciences®, Leipzig, Germany SP947257). Samples were then tested by real-time PCR for the following bacteria: *Francisella tularensis* (42), *Borrelia* spp. (43), *Rickettsia* spp. (44), *Coxiella burnetii* (45), *Anaplasma* spp., *Ehrlichia* spp. and *Bartonella* spp. (46). For real-time PCR targeting *Borrelia* spp., a field-positive control corresponding to *Borrelia burgdorferi* was used and samples with a Ct value below 38 were considered positive. For *Rickettsia* spp., a field-positive control corresponding to *Rickettsia helvetica* was used, and samples with a Ct value below 38 were considered positive. For *Bartonella* spp., a field-positive control corresponding to *Bartonella henselae* was used, and, likewise, samples with a Ct value below 38 were considered positive. For all PCR assays, a negative amplification control consisting of 5 μL of DNase/RNase-free water was included. Moreover, the following viruses were investigated using real-time PCR: Tick Borne Encephalites Virus (47), Crimean Congo Haemorragic Fever (48), West Nile Virus (49, 50), Usutu virus (51), and phleboviruses (52). Rickettsia species identification was performed by sequencing the gltA gene fragment. Samples testing positive for *Borrelia* spp. were further analysed by endpoint PCR targeting the *Borrelia burgdorferi* sensu lato complex and subsequently screened for *B. afzelii* in positive cases. Samples negative for *B. afzelii* were then subjected to sequencing for species-level identification. The positive amplicons were purified using the BigDye™ Terminator v3.1 Cycle Sequencing Kit (Applied Biosystems™, Thermo Fisher Scientific, Monza, Italy) and sequenced (SeqStudio Genetic Analyser, Applied Biosystems™, Thermo Fisher Scientific, Monza, Italy). Sequence quality was assessed by visual inspection of the obtained sequences using BioEdit software v. 7.2.5 and by examination of the corresponding electropherograms. The sequences were then compared with those available in the NCBI GenBank database using the BLAST tool.

Laboratory personnel were not formally blinded to sample identity during molecular analyses. Samples were processed using unique laboratory identification codes, and molecular assays were performed according to standardised protocols and predefined criteria for pathogen detection.

### 2.5 Statistical analysis

The data were exported to an Excel spreadsheet (see Supplementary material), and the ecological indices (prevalence, intensity, mean intensity, and mean abundance) for ticks were measured according to Bush et al. (53). The statistical analyses were carried out using RStudio (version 2024.4.2.0). The normality of the quantitative variables was checked using the Shapiro–Wilk test. Differences in *R. sanguineus* tick counts among months were evaluated using the Kruskal–Wallis test. Spearman’s rank correlation coefficient was used to assess associations between tick abundance and environmental variables, including altitude, temperature, humidity, and rainfall.

## 3 Results

Among the 44 Cape hare individuals examined, 22 (50%, CI 95%: 35.8–64.2) were found to be infested with ticks, whereas none of the wild rabbits harboured ticks. A total of 165 ticks (56 pools) were collected, with *R. sanguineus* sensu lato (sl) being the predominant species (94.5%; 156/165), followed by *I. hexagonus* (5.5%; 9/165).

A total of nine tick specimens were submitted to MALDI-TOF MS analysis to confirm the morphological identification. Of these, three samples yielded negative results, as no protein spectra were detected. One Rhipicephalus specimen was reliably identified as *R. sanguineus* (score: 2.15), while the remaining samples had low-confidence or inconclusive identifications (score <1.8). For this reason, it was decided to maintain the morphological identification, therefore *R. sanguineus* sl.

Adult ticks were the most prevalent stage, followed by nymphs, while larvae were rarely encountered. Co-infestation by multiple developmental stages of *R. sanguineus* sl was seen in some hares, including nymph/adult combinations in four cases (8.5%) and the simultaneous presence of larvae, nymphs, and adults in one case (2.1%). All *I. hexagonus* specimens were identified as adults (three males and four females), or nymphs (*n* = 2), with no larvae detected. (Table 2).

**Table 2:** Prevalence, mean abundance, intensity, and mean intensity of ticks in infested hares *Lepus capensis*, from Algeria.

Ticks	No and rate	Prevalence (%)	Mean Abundance	Intensity	Mean Intensity
*Rhipicephalus sanguineus sl*	**156/165**	**94.5**	**3.31**	**1–62**	**7.09**
Adults	123/156	78.8	2.61	1–45	5.85
Females	79/156	50.6	1.68	1–44	6.07
Males	44/156	28.2	0.93	1–11	2.75
Nymphs	29/156	18.6	0.61	1–13	5.8
Larvae	4/156	0.03	0.08	0–4	4.0
*Ixodes hexagonus*	**9/165**	**5.4**	**0.19**	**1–6**	**1.8**
Adults	7/9	78	0.14	1–2	1.4
Females	4/9	44.4	0.08	0–1	1.0
Males	3/9	33.3	0.06	1–2	1.5
Nymphs	2/9	22.2	0.04	0–2	2.0
Larvae	0/9	0	0	0	0

The values in bold refer to the total number of ticks identified, *Rhipicephalus sanguineus* sl and *Ixodes hexagonus* respectively.

The infestation rate varied seasonally, peaking in September at 100% of affected hares with a mean tick burden of 11.57 ± 22.37 (maximum = 62). In October and November, values declined to 40.9 and 46.2%, respectively, with lower mean tick counts (2.27 ± 3.77 in October and 1.82 ± 3.52 in November). No ticks were detected in December (Table 3).

**Table 3:** Comparison of *Rhipicephalus sanguineus* tick counts (n_ticks) across different months using the Kruskal–Wallis test.

Months	Examined hares	Infeasted hares	Mean	Median	Min	Max	Standard deviation	Prevalence %	CI 95%
Sep	7	7	11.57	2	2	62	22.37	100^a^	64.6–100
Oct	21	9	2.27	0	0	16	3.77	40.9^b^	23.3–61.3
Nov	11	6	1.82	0	0	12	3.52	46.2^b^	23.2–70.9
Dec	5	0	0.00	0	0	0	0.00	0^c^	0–43.4

^a,b,c^Significant differences can be seen between different superscripts within the same row.

CI, Confidence Interval.

Molecular analysis revealed the presence of several zoonotic pathogens (Table 4). Specifically, tick-borne pathogens were detected in ticks from 10 of the 22 infested hares (45.4%, CI 95%:26.9–65.3). Among these, *Rickettsia* spp. was identified in 36.4% (CI 95%: 19.7–57) of examined individuals (8/22), while *Borrelia* spp. was detected in 13.6% (CI 95%: 4.7–33.3 -3/22).

**Table 4:** Percentage of pools positive to *Rickettsia* and *Borrelia* species detected in ticks collected from *Lepus capensis* in Algeria.

Genus	Species	*Rhipicephalus sanguineus*			*Ixodes hexagonus*			Total		
		*n*/tot	*p* (%)	CI 95%	*n*/tot	*p* (%)	CI 95%	*n*/tot	*p* (%)	CI 95%
*Rickettsia*	*R. aeschlimanii*	11/50	22%	12.7–35.2	0/6	0%	–	11/56	19.64%	11.3–31.8
	*R. massiliae*	3/50	6%	2–16.2	0/6	0%	–	3/56	5.35%	1.8–14.6
	*R. sibirica*	2/50	4%	1.1–13.4	0/6	0%	–	2/56	3.57%	0.9–12.1
	*R. africae*	1/50	2%	0.3–10.5	0/6	0%	–	1/56	1.78%	0.3–9.4
	*R. raoltii*	1/50	2%	0.3–10.5	0/6	0%	–	1/56	1.78%	0.3–9.4
	*R. japonica*	1/50	2%	0.3–10.5	0/6	0%	–	1/56	1.78%	0.3–9.4
	*R. marmionii*	1/50	2%	0.3–10.5	0/6	0%	–	1/56	1.78%	0.3–9.4
	*Rickettsia spp*	6/50	12%	5.6–23.8	0/6	0%	–	6/56	10.71%	0.5–21.4
*Borrelia*	*B. afzelii*	1/50	2%	0.3–10.5	1/6	2%	3–56.3	2/56	3.57%	0.9–12.1
	*B. burgdorferi*	1/50	2%	0.3–10.5	0/6	0%	–	1/56	1.78%	0.3–9.4
	*Borrelia spp*	1/50	2%	0.3–10.5	0/6	0%	–	1/56	1.78%	0.3–9.4

Considering the 56 tick pools analysed (50 pools of *R. sanguineus* and 6 pools of *I. hexagonus*), *Rickettsia* spp. was found in 26 pools (46.4%, CI 95%:34–59.3) and *Borrelia* spp. in 4 pools (7.1%, CI 95%: 2.8–16.9).

Molecular identification revealed 7 Rickettsia species and 2 Borrelia species. However, 6 Rickettsia-positive samples (10.7%; 6/56) and one Borrelia-positive sample (1.8%; 1/56) could not be identified to the species level. *Rickettsia* spp. was detected exclusively in *R. sanguineus* sl pools and was absent in *I. hexagonus* pools. Among the identified species, *R. aeschlimannii* was the most prevalent (19.6%), followed by *R. massiliae* (5.3%) and *R. sibirica* (3.6%). Other species, including *R. africae*, *R. japonica*, *R. marmionii*, and *R. raoultii*, were detected at low prevalence (1.8%).

Zoonotic Borrelia was detected in one of 50 *R. sanguineus* pools (2%) and in one of six *I. hexagonus* pools (16.7%). Borrelia species identified included *Borrelia afzelii* (3.6%), detected both in *R. sanguineus* sl and *I. hexagonus*, and both *Borrelia burgdorferi* sensu stricto (1.8%) and *Borrelia* spp. (1.8%) in *R. sanguineus* sl.

Six samples positive for Rickettsia spp. and one sample positive for Borrelia spp. could not be resolved to the species level by sequencing and were therefore reported as *Rickettsia* spp. and *Borrelia* spp., respectively.

Two co-infections were identified among the analysed tick pools: one involving 5 Rickettsia species (*R. africae*, *R. japonica*, *R. marmionii*, *R. raoultii*, and *R. sibirica*) together with *Borrelia burgdorferi*, and another involving *R. aeschlimannii* and *Borrelia* spp. (2/56 = 3.6%).

All ticks tested were negative for *Anaplasma* spp., *Coxiella burnetii*, *Ehrlichia* spp., and the screened viral agents (TBEV, CCHF, WN, USUTU, and phleboviruses).

Pathogen detection rates were expressed as the proportion of positive pools among all pools tested. Because ticks were analysed individually or in pools of up to six specimens, these values represent pool-level detection rates and should not be interpreted as individual-tick infection prevalence.

**Figure 2:**
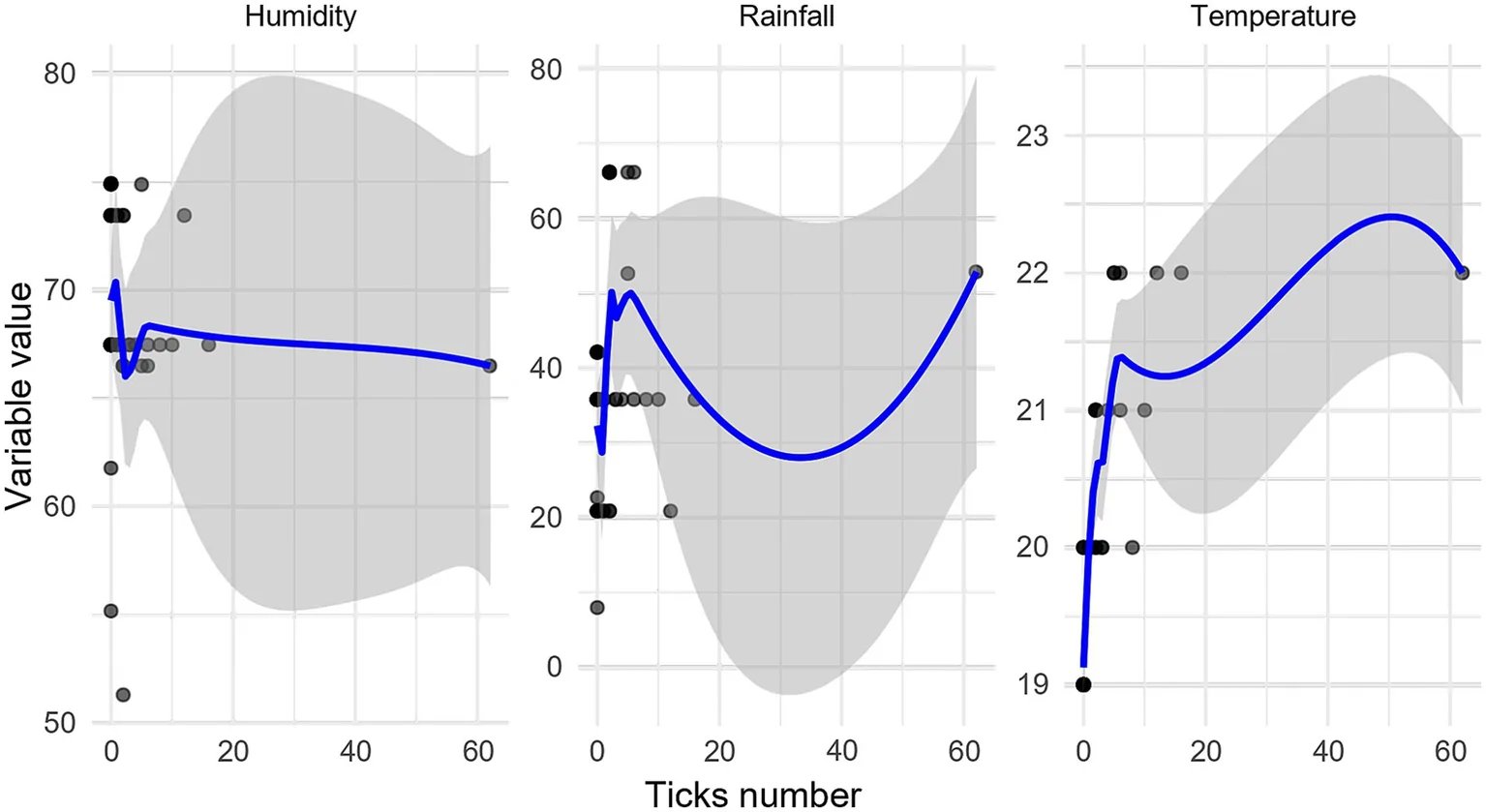
Relationships between tick counts and climatic parameters.

Statistical analysis revealed significant temporal variation in *R. sanguineus* sl infestation (Kruskal–Wallis *H* = 9.86, df = 3, *p* < 0.05). Tick abundance showed a positive correlation with temperature (rs = 0.370, *p* = 0.011) and negative correlations with humidity (rs = −0.312, *p* = 0.033) and rainfall (rs = −0.305, *p* = 0.038). No significant association was observed with altitude (rs = 0.120, *p* = 0.423) (Figure 2).

## 4 Discussion

This study provides the first comprehensive assessment of tick infestations and associated zoonotic pathogens in Cape hare populations in Algeria. The study was therefore designed primarily as an exploratory investigation to characterise tick infestation and identify associated bacterial and viral pathogens, rather than to provide definitive population-level estimates of pathogen prevalence. The resulting sample size was considered appropriate for generating baseline information on tick–host–pathogen associations in the study area, while recognising that larger studies will be required to confirm these findings and provide more precise estimates of prevalence and seasonal patterns.

Given the aim of the study and the relatively limited sample size, at this stage it was decided not to investigate further relationships between the ticks’ infestation status and the morphobiometric data of individual animals. A previous study carried out in Kenya demonstrated a link between the sex and age of hares and the presence of ticks, suggesting that adult males are more likely to be infested with ticks (54). However, studies exploring these aspects are very limited, and it would be worthwhile to investigate them in depth using a larger sample size. *R. sanguineus* sl was the dominant tick species infesting Cape hares, about half harbouring at least one tick, whereas *I. hexagonus* was only sporadically detected. As expected, adult ticks represented the most prevalent developmental stage. The infestation rate observed in this study was higher than that reported in Spain for both European brown hare (14–31%) (55, 56) and Iberian hare (22–42%) (57, 58), but lower than that reported in wild rabbits from Sicily (75.2%) (59).

The predominance of *Rhipicephalus sanguineus* sl observed in the present study may reflect the combined influence of host availability, habitat characteristics, and environmental conditions. *R. sanguineus* sl is highly adaptable and is primarily associated with domestic dogs, but it can also infest a broad range of wild and domestic mammals, facilitating its persistence in areas where different host species coexist or overlap. Its ability to survive in sheltered and peridomestic environments may further contribute to its establishment and persistence under suitable conditions (9). In Algeria, *R. sanguineus* sl has been reported from several domestic and wild hosts, supporting its broad ecological plasticity (60). Climatic factors, particularly temperature and humidity, may also influence its development, survival, and seasonal activity, and differences in climatic conditions have been associated with the distribution of lineages within the *R. sanguineus* complex (61). The predominance of this tick species on Cape hares may therefore reflect the availability of suitable hosts and favourable environmental conditions, potentially enhanced by interactions between wildlife and domestic or peridomestic environments. However, these factors were not directly quantified in the present study and should therefore be considered plausible explanations rather than demonstrated determinants of tick abundance.

Regarding the tick species identified, *R. sanguineus* and *I. hexagonus*, our findings are consistent with previous studies reporting *R. sanguineus* as the predominant tick species infesting domestic dogs in semi-arid and arid bioclimatic regions of Algeria (62, 63). Although dogs are their primary hosts, *R. sanguineus* exhibits considerable ecological plasticity and can parasitize a wide range of vertebrate hosts, including wildlife species (9).

An integrated approach combining morphological examination and MALDI-TOF MS analysis was attempted for species identification. While morphological identification allowed for reliable species-level assignment, MALDI-TOF mass spectrometry was applied in an attempt to confirm the species, particularly in morphologically complex groups such as *R. sanguineus*.

The *R. sanguineus* group (*R. sanguineus* sl) includes morphologically and phylogenetically closely related species (38).

Despite recent advances, its taxonomy remains challenging, mainly due to the limited availability of reference DNA sequences in public databases (39). In this context, MALDI-TOF MS has proven to be a reliable and complementary tool for tick identification, helping to resolve taxonomic ambiguities within the *R. sanguineus* group and closely related species such as *R. pusillus*. Similarly, although *I. hexagonus* is considered a specialist parasite (64), it is also routinely observed in several other small mammalian hosts (65). However, in the present study, MALDI-TOF MS analysis did not yield conclusive results for this species, with identification scores remaining below the reliability threshold (score < 1.8). This outcome was likely attributable to prolonged storage in ethanol, which has been shown to cause progressive deterioration of spectral quality. (41, 66–70).

The seasonal trend of infestation, i.e., a peak in early autumn, is consistent with the known activity patterns of *R. sanguineus*, which is most active under warmer temperatures and reduced humidity and becomes largely inactive during colder, wetter months. A previous study showed that this tick is adapted to semi-arid (Souk-Ahras) and arid (Tebessa) climates, characterised by high temperatures, low humidity and rainfall (71, 72). Consistently, our statistical analyses revealed a positive association between temperature and tick abundance, and negative associations with humidity and rainfall, confirming the strong influence of climatic factors on tick population dynamics (73, 74).

The predominance of *R. sanguineus* in Cape hares is epidemiologically important because it is a competent vector for multiple zoonotic agents, including those detected in this study. The detection of 7 Rickettsia species, all within the spotted fever group (SFG), aligns with recent studies in Algeria, which report *R. aeschlimanii* in several tick species from both domestic and wild animals, but not in *R. sanguineus* (14, 26, 75, 76). Nevertheless, in other countries, *R. aeschlimanii* was also the most common pathogen in *R. sanguineus* collected from domestic and wild animals, with varying prevalence (77).

In addition, our findings extend the known host range of *R. massiliae* and *R. aeschlimannii*, previously detected in ticks from domestic and wild animals in Algeria, suggesting that wild lagomorphs may contribute to the maintenance of these pathogens in natural ecosystems (62, 63, 75, 76).

The detection in *R. sanguineus* of *R. sibirica*, the causative agent of lymphangitis-associated rickettsiosis (LAR) (78), is noteworthy, as this species has previously been associated primarily with Hyalomma ticks (79–80). The detection of *R. africae*, responsible for African tick-bite fever, and *R. japonica*, the etiological agent of Japanese spotted fever, historically associated, respectively, with *Amblyomma* spp. and *H. dromedarii* (81, 82) and confined to the sub-Saharan African region (83), suggests possible ecological shifts, pathogen expansion, or introduction through wildlife movement or climate-mediated range changes.

The detection of multiple Rickettsia species with zoonotic potential highlights their potential public health relevance in Algeria. Previous studies have documented the circulation of *R. conorii*, *R. aeschlimannii,* and *R. massiliae* in Algerian ticks, while human infections with *R. aeschlimannii* have also been reported in the country (20, 75). These findings indicate that several zoonotic rickettsiae circulate within local tick populations and may be relevant to human health. However, the detection of Rickettsia DNA in ticks does not necessarily indicate viable pathogens or demonstrate transmission to humans. The potential public health impact depends on several interacting factors, including tick abundance, vector competence, pathogen prevalence and viability, host availability, and human exposure to infected ticks. Therefore, our findings should be interpreted as evidence of the circulation of potentially zoonotic Rickettsia rather than as a direct measure of human infection risk. Nevertheless, the identification of multiple zoonotic Rickettsia species supports the need for integrated surveillance combining molecular investigation of ticks with epidemiological surveillance of human rickettsioses, particularly at the wildlife–domestic animal–human interface.

Zoonotic Borrelia species were identified for the first time in both *R. sanguineus* and *I. hexagonus* collected from Cape hares in Algeria. Although *Borrelia burgdorferi* sensu lato and *B. afzelii* have previously been reported in European lagomorphs (23, 84), their detection in this context suggests that Cape hares may contribute to the maintenance of Lyme borreliosis agents in natural environments. However, their precise role as reservoir hosts and their contribution to pathogen transmission cycles require further investigation. The detection of ticks and tick-borne pathogens associated with Cape hares suggests that these animals may be involved in local tick–host–pathogen interactions. However, their epidemiological role should be interpreted cautiously, as the present study did not assess reservoir competence, host infectiousness, pathogen persistence, or transmission dynamics. Therefore, the detection of pathogen DNA or RNA in ticks collected from Cape hares does not demonstrate that these animals act as competent reservoirs or contribute to pathogen maintenance or transmission. Further longitudinal and experimental studies are needed to clarify the potential role of Cape hares in the ecology and circulation of tick-borne pathogens.

### 4.1 Limitations

Several limitations should be considered when interpreting these findings. The sample size (44 Cape hares and 3 rabbits) was largely determined by wildlife availability and the practical and ethical constraints of sampling free-ranging animals. Therefore, the study was primarily exploratory and was not intended to provide definitive population-level estimates of pathogen prevalence and/or statistically validated information. Pooling ticks may also reduce the precision of infection estimates, as positive pools indicate pathogen occurrence but do not allow individual-tick prevalence to be determined. Six Rickettsia-positive and one Borrelia-positive samples could not be identified to species level, possibly due to technical limitations such as low pathogen DNA concentration or insufficient sequencing quality; these samples were therefore conservatively reported as *Rickettsia* spp. and *Borrelia* spp. Finally, the restricted geographical coverage and single-year sampling limit the generalisability of the findings. Larger, multi-site, and multi-year studies based on individual-tick testing are needed to confirm these results and better assess spatial and temporal variation in tick infestation and pathogen circulation.

## 5 Conclusion

Our study identified *Rhipicephalus sanguineus* as the predominant tick species infesting Cape hares in the High Plateaus of Algeria. This species can harbour multiple zoonotic pathogens of public health concern, including Rickettsia and Borrelia. Nevertheless, further investigations are needed to better understand the role of wild lagomorphs and their ticks in the transmission of zoonotic diseases that threaten public health. Including hares in tick-borne disease surveillance programs may help understand the ecology of ticks and their pathogens in Cape hares and design One Health strategies to prevent the spread of tick-borne diseases to humans and domestic animals in Algeria and the wider North African region.

## Data Availability

The original contributions presented in the study are included in the article/Supplementary material, further inquiries can be directed to the corresponding authors.

## Supplementary material

SUPPLEMENTARY TABLE S2 Data collected from hunted animals, including morphobiometric information, data about the presence of ticks and related pathogens.
